# Zoltan Lukacs Passed Away

**DOI:** 10.3390/ijns6040086

**Published:** 2020-11-03

**Authors:** Joseph Orsini, Michael Gelb, Peter C. J. I. Schielen

**Affiliations:** 1 Wadsworth Center, New York State Department of Health, Newborn Screening Program, David Axelrod Institute, 120 New Scotland Ave., Albany, NY 12201, USA; joseph.orsini@health.ny.gov; 2 Department of Chemistry, University of Washington, Seattle, WA 98195, USA; gelb@uw.edu; 3 Department of Biochemistry, University of Washington, Seattle, WA 98195, USA; 4 Office of the International Society for Neonatal Screening, Reigerskamp 273, 3607 HP Maarssen, The Netherlands

It is with great sadness that we have to inform you of the passing on 13 August 2020, of Dr. Zoltan Lukacs (Figure 1).

Dr. Zoltan Lukacs was born in Hungary (1972) and received his scientific training in Germany, mainly in the intersection of inorganic, organic, and analytical chemistry. He received his Ph.D. degree at the University of Würzburg (2000). He was appointed head of the neonatal screening and metabolic laboratory in the pediatric department of the Hamburg University Hospital. For additional training, he spent some time in 2004 working with Genzyme (Boston, MA, USA) and at Duke University (Durham, NC, USA). Zoltan worked on the application of Luminex technology in neonatal screening, and subsequently shifted his attention to the study of lysosomal storage diseases. In 2003, he made available a quality assurance programme for amino acids and acylcarnitines via MS/MS, with worldwide participation. In addition, he successfully developed links with a large number of screening centres in developing countries, helping to support and develop these services.

During his education, Zoltan received a number of awards, such as the Würzburg University Faculty prize for his Ph.D. thesis. He was awarded the first ISNS/Bio-Rad Dussault Medal for young investigators in 2007 for his pioneering activities in newborn screening.

Between 2007 and 2019, Zoltan expanded his expertise and specialties, particularly in the field of lysosomal storage diseases. He hosted, amidst the European COVID-19 lockdown, the meeting of the European Pompe Consortium earlier this year.

He also expanded his work for developing countries. In 2019, he was nominated for the ISNS/Labsystems Gerard Loeber award, a new ISNS award for distinguished contributions to newborn screening expansion in developing countries. This was certainly where Zoltan’s heart lay, in supporting and fostering emerging programmes using his insight and knowledge of newborn screening, its primary process, and quality assurance.

In Ecuador, Zoltan was involved in a cooperation to provide an extended range of newborn screening services with the Instituto Andino des Enfermedades Metabolicas. He gave numerous lectures and advised on implementation. This cooperation was still ongoing in 2019, with the ultimate goal of improving newborn screening in Ecuador.

Zoltan was also instrumental in setting up the extended newborn screening program in Lebanon. This provided the local population with better access to newborn screening services. Other efforts around the world to support and build newborn screening programs included Iceland; Uzbekistan; Guangdong Province, China; Mongolia; Kazakhstan; Madagascar; and Laos. His focus was always on empowering local programs and allowing a sustainable local screening program to develop. Recently, in Laos, this has resulted in a new local screening effort, carried out by national staff and supported by Dr. Lukacs and Dr. Thomas Hoehn.

Newborn screening is a small world, and newborn screening for lysosomal storage disorders (LSDs) an even smaller one. Zoltan welcomed old and new colleagues in the field with open arms, and some of us quickly became not only colleagues but friends as well. 

Zoltan was a valued partner in setting up screening for LSDs in Albany, NY, United States, where he also worked as a visiting specialist for some time. Zoltan was always willing to help better understand the biochemistry of the LSD disorders. His love for life and science was evident, as was his passion for improving newborn screening.

Zoltan’s scientific output was impressive—he was the (co-)author of over 120 publications.

Zoltan was a very active and dedicated ISNS member in so many ways. He served as member of the ISNS Council form 2009–2013 and was again elected in 2019 for the term 2019–2022.

He was a member of the local organizing committee of the 2021 ISNS conference in Luxemburg, and we will greatly miss his unique contributions to that conference and in so many other ways. He was a visionary within the newborn screening world, and dared to think creatively for the future.

Apart from all that, Zoltan was an absolutely great guy to have around. In his signature outfit, a polo shirt and his bag worn with the strap crossed on his chest, he would, with a charming hint of a German accent, thoughtfully comment on presentations. He felt comfortable discussing both the complex analytical details, as well as clinical applications. After congress hours, he was that same beloved colleague, happy to chat about more mundane subjects, such as the beers of Hamburg, which was where he lived with his family.

Zoltan had all the potential to become one of the guardians of international newborn screening for decades to come, a prospect now stopped short by his untimely death, the consequences of a COVID-19 infection.

Our heart goes out to his wife Paulina and children Elizabeth and Anthony for their awful loss.

His colleagues will cherish his memory and miss his intelligence, his insight, his smile, and his friendship.

## Figures and Tables

**Figure 1:**
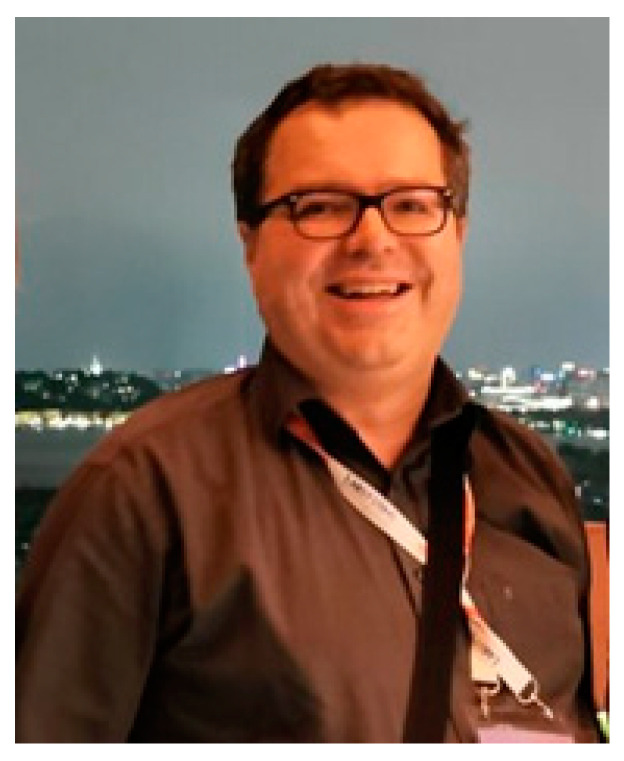
Zoltan at the congress dinner at the 10th International Conference on neonatal screening in Hangzhou, China, September 2019.

